# Juvenile polyposis syndrome: A case report

**DOI:** 10.1016/j.ijscr.2019.04.041

**Published:** 2019-05-03

**Authors:** A. Pérez-Castilla, P. Peñailillo, D. Oksenberg

**Affiliations:** a Department of Digestive Surgery, Clinica Indisa. Santiago, Chile; b Department of gastroenterology, Clinica Indisa. Santiago, Chile; c University Andrés Bello, Faculty of Medicine. Santiago, Chile

**Keywords:** Total gastrectomy, Juvenile polyposis, Endoscopic polypectomy

## Abstract

**Introduction:**

Juvenile polyposis syndrome it is an uncommon autosomal dominant inherited condition. Hamartomatous polyps can affect the entire gastrointestinal tract but usually predominates in the colon. We introduce a case of juvenile polyposis syndrome presented with massive gastric polyposis that requires a total gastrectomy.

**Case presentation:**

A 22-year-old man presented symptoms of chronic upper gastrointestinal bleeding. Gastroscopy showed massive gastric polyposis. Initially endoscopic polypectomy was performed, but due to the progressive symptoms, a total gastrectomy was then performed. Histology confirmed massive gastric juvenile polyposis.

**Conclusion:**

Massive gastric polyposis it is an uncommon manifestation of juvenile polyposis syndrome.

## Highlights

• The development of polyps is a rare genetic defect.
• juvenile polyposis syndrome are at greater risk of colorectal and gastric cáncer.
• The only treatment to avoid the risk of malignancy is total resection.

## 1 Introduction

Juvenile polyposis syndrome (JPS) it is an autosomal dominant condition characterized by multiple hamartomatous polyps. Individuals with JPS are at greater risk of colorectal and gastric cancer [1,2]. In contrast to JPS, sporadic juvenile polyps, are not associated with an increased cancer risk [3]. This case report, shows an unusual case of juvenile polyposis syndrome with massive gastric polyposis that requires a total gastrectomy.

The work has been reported in line with the SCARE criteria [7].

## 2 Case

**Fig. 1:**
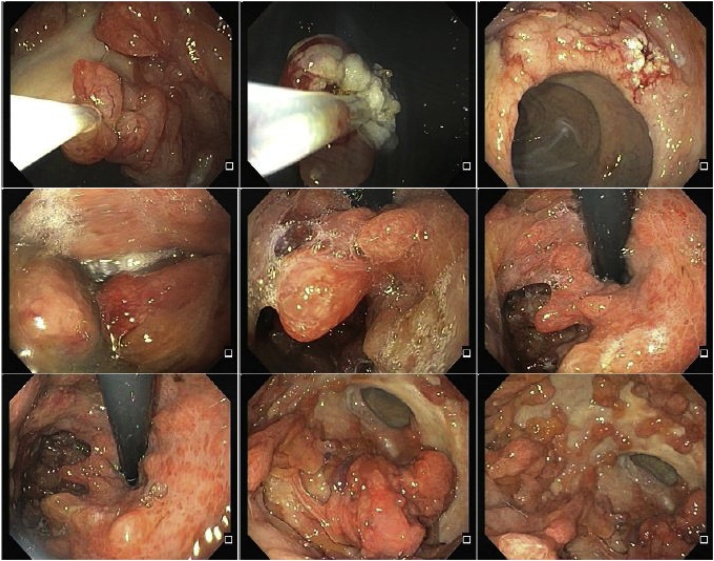
ENDOSCOPY: Juvenile polyposis with diffuse gastric involvement, with spontaneuos bleeding of some lesión, without pyloric obstruction.

**Fig. 2:**
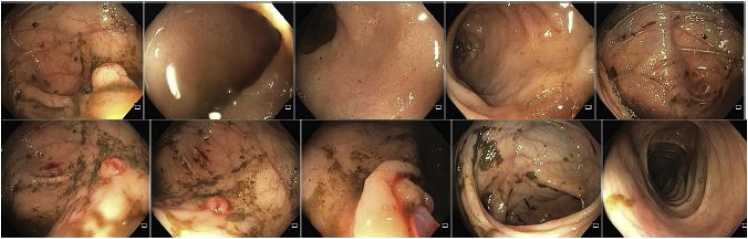
COLONOSCOPY: Resected sessile cecal polyps.

**Fig. 3:**
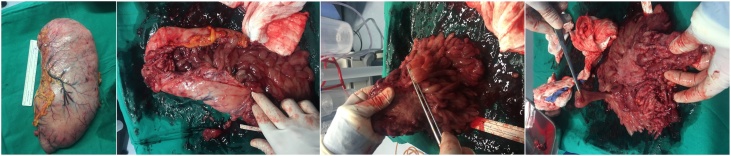
TOTAL GASTRECTOMY. MASSIVE GASTRIC POLYPOSIS. MASSIVE GASTRIC POLYPOSIS. MASSIVE GASTRIC POLYPOSIS.

A 22-year-old man presented symptoms of chronic upper gastrointestinal bleeding. Endoscopy (Fig. 1) showed massive gastric polyposis, while colonoscopy showed a few polys (Fig. 2). At first, endoscopic polypectomy was executed, but due to the progressive symptoms, a total gastrectomy was then performed (Fig. 3). Histology confirmed massive gastric juvenile polyposis.

## 3 Discussion

Juvenile polyposis syndrome (JPS) it is an autosomal dominant condition characterized by multiple hamartomatous polyps. Individuals with JPS are at greater risk for colorectal and gastric cancer [1,2]. In contrast to JPS, sporadic juvenile polyps, are not associated with an increased cancer risk [3].

Polyps usually begin to appear in the first decade of life and occur predominantly in the colorectum (98%), stomach (14%), duodenum (7%), jejunum, and ileum (7%).

Individuals with JPS are considered to be at an increased risk for colorectal cancer. The cumulative risk of colorectal cancer in individuals with JPS is from 17% to 22% [5,6]. Individuals with JPS are also at an increased risk for gastric cancer, with an estimated lifetime risk from 20% to 30% and a mean diagnosis age of 58 years [1,4,5].

A clinical diagnosis is based on the presence of at least one of the following criteria and the absence of clinical manifestations of other hamartomatous polyposis syndromes [1,4] : 1) More than five juvenile polyps in the colorectum, 2) Multiple juvenile polyps in other parts of the gastrointestinal tract and 3) Any number of juvenile polyps in a person with a known family history of juvenile polyps.

Individuals who meet clinical criteria for JPS should undergo genetic testing for a germline mutation in the *BMPR1A* and *SMAD4*genes. Genetic testing in an individual who meets clinical criteria for JPS serves to confirm the diagnosis of JPS and to counsel at-risk family members.

Complete or partial gastrectomy may be necessary for patients with advanced dysplasia, gastric cancer, or massive gastric polyps that cannot be effectively managed whit endoscopic.

## 4 Conclusion

Juvenile polyposis syndrome is an inherited disease, so it is not possible to prevent it. This case report illustrates the importance of making the diagnosis of juvenile polyposis syndrome. People with gastric polyposis should be treated initially with endoscopic polypectomy, however a total gastrectomy may sometimes be necessary.

## Conflicts of interest

No conflicts of interest.

## Sources of funding

We no uses any sources of funding.

## Ethical approval

This study is exempt from ethnical approval in our institution.

## Consent

Written informed consent was obtained from the patient for publication of this case report and accompanying images. A copy of the written consent is available for review by the Editor-in-Chief of this journal on request.

## Author contribution

- First author, Alberto Pérez-Castilla: Design study, data analysis, data collection.
- Danny Oksenberg: Design study.
- Pablo Peñailillo: Data collection, writing the paper

## Registration of research studies

Unique identifying number: 4608.

## Guarantor

Alberto Pérez Castilla.

## Provenance and peer review

Not commissioned, externally peer-reviewed.
